# Transcriptome Sequencing of Mung Bean (*Vigna radiate* L.) Genes and the Identification of EST-SSR Markers

**DOI:** 10.1371/journal.pone.0120273

**Published:** 2015-04-01

**Authors:** Honglin Chen, Lixia Wang, Suhua Wang, Chunji Liu, Matthew Wohlgemuth Blair, Xuzhen Cheng

**Affiliations:** 1 The National Key Facility for Crop Gene Resources and Genetic Improvement, Institute of Crop Science, Chinese Academy of Agricultural Sciences, Beijing, China; 2 CSIRO Plant Industry, Queensland Bioscience Precinct, Queensland, Australia; 3 Department of Agricultural and Environmental Sciences, Tennessee State University, Nashville, Tennessee, United States of America; National Institute of Plant Genome Research, INDIA

## Abstract

Mung bean (*Vigna radiate* (L.) Wilczek) is an important traditional food legume crop, with high economic and nutritional value. It is widely grown in China and other Asian countries. Despite its importance, genomic information is currently unavailable for this crop plant species or some of its close relatives in the *Vigna* genus. In this study, more than 103 million high quality cDNA sequence reads were obtained from mung bean using Illumina paired-end sequencing technology. The processed reads were assembled into 48,693 unigenes with an average length of 874 bp. Of these unigenes, 25,820 (53.0%) and 23,235 (47.7%) showed significant similarity to proteins in the NCBI non-redundant protein and nucleotide sequence databases, respectively. Furthermore, 19,242 (39.5%) could be classified into gene ontology categories, 18,316 (37.6%) into Swiss-Prot categories and 10,918 (22.4%) into KOG database categories (E-value < 1.0E-5). A total of 6,585 (8.3%) were mapped onto 244 pathways using the Kyoto Encyclopedia of Genes and Genome (KEGG) pathway database. Among the unigenes, 10,053 sequences contained a unique simple sequence repeat (SSR), and 2,303 sequences contained more than one SSR together in the same expressed sequence tag (EST). A total of 13,134 EST-SSRs were identified as potential molecular markers, with mono-nucleotide A/T repeats being the most abundant motif class and G/C repeats being rare. In this SSR analysis, we found five main repeat motifs: AG/CT (30.8%), GAA/TTC (12.6%), AAAT/ATTT (6.8%), AAAAT/ATTTT (6.2%) and AAAAAT/ATTTTT (1.9%). A total of 200 SSR loci were randomly selected for validation by PCR amplification as EST-SSR markers. Of these, 66 marker primer pairs produced reproducible amplicons that were polymorphic among 31 mung bean accessions selected from diverse geographical locations. The large number of SSR-containing sequences found in this study will be valuable for the construction of a high-resolution genetic linkage maps, association or comparative mapping and genetic analyses of various *Vigna* species.

## Introduction

Mung bean belongs to the *Vigna* genus within the *Phaseoleae* tribe and is a diploid crop (2n = 2x = 22) with a genome size of approximately 560 Mb. It is widely grown in Asia as an important nutritional dry grain, food legume pulse that is complementary to rice for the balanced nutrition it provides to millions of people across China, Cambodia, Laos and Vietnam, to name a few of the countries where the crop is grown. Mung bean is thought to have health promoting and nutritional characteristics in the diet and can be used to improve soil fertility given high rates of nitrogen fixation [1]. In addition, mung bean vegetable sprouts are popular in Asian cuisine, and they are good sources of protein, fiber, vitamin C and minerals.

Studies in genetic diversity, map-based cloning and molecular breeding of mung bean have lagged behind other legume crops due to the lack of genomic information for this pulse crop species [2]. Previous efforts to develop molecular markers for mung bean have not generated sufficient markers for linkage saturated map construction because they were either monomorphic or not fully informative for bi-parental mapping populations. This has been the case for RFLP [3], RAPD [4], AFLP [5], CAPS [6] and SNP [7] markers. Therefore, microsatellites (also known as SSRs based on their Simple Sequence Repeat core) are a logical choice for broadening the scope of markers available to mung bean researchers.

A promising source of SSR markers for legumes is found in the EST sequences generated by traditional or full transcriptome evaluation techniques. Sanger sequence ESTs in legumes as in other crops have for the most part contained an abundance of SSR repeat types [8]. With up-to-date technology, next generation sequencing of the transcriptome, a method often called RNA-seq, provides deeper and broader coverage of the transcriptome than traditional Sanger sequencing [9]. In addition, RNA-seq provides a lower background to signal ratio, better coverage of adenylation signals and a larger dynamic range of gene expression levels for mRNA evaluation than previous sequencing methods [10,11].

RNA-seq technology has been successfully and ubiquitously applied to both model and non-model organisms [12–14]. In mung bean, transcriptome studies have been limited and to date only 454 FLX rather than Illumina sequencing has been used with this species. Therefore, the overall goal of this research was to conduct transcriptome analysis with RNA-seq and to obtain usable EST-SSR markers from sequencing with Illumina technology. There are few reports on the development of SSR markers in mung bean to date. Somta et al. (2011) designed 157 genic microsatellite markers in mung bean but these were of low polymorphism [15]. In follow up studies, Moe et al. (2011) identified 1,630 SSR loci from mung bean mRNAs of the genotype Jangan derived from 454 sequencing technology [16]; and Gupta et al (2014) designed 1,742 SSR markers from EST sequences of the same variety [17]. However, far fewer SSR markers are reported in mung bean than in common bean [8,18], chickpea [19–20], pigeon pea [21] and soybean [22].

The full objective of this study was to use RNA-seq technology and Illumina based transcriptome evaluation of two mung bean cultivars to develop EST-based SSRs for the crop given the low number of markers available for the species. We characterize the distribution of SSR motifs in the sequences generated and validate a group of EST-SSR for further use in diversity analysis. We discuss the utility of the microsatellite markers for comparative mapping.

## Materials and Methods

### Plant material

A total of 33 mung bean accessions were used in this study including the varieties, ZL1 and V6, for RNA-seq and an additional 31 accessions for genetic diversity analysis (S1 Dataset). Of the 31 accessions, 8 genotypes were used for initial screening and validation of marker polymorphism. These mung bean accessions were obtained from the National Center for Crop Germplasm Resources Preservation located in Institute of Crop Science, Chinese Academy of Agricultural Sciences, Beijing, China and were grown for DNA and RNA extractions in a research field at the same location.

### RNA extraction

Tissue samples of roots, stems and leaves were collected at 15 days after sowing and quickly frozen in liquid nitrogen for storage at -80°C. RNA from each of the samples was isolated using the Trizol Reagent with manufacturer’s instructions (Invitrogen, Life Technologies, Carlsbad, USA). Total RNA was then treated with RNase-free DNase I (Takara, Kyoto, Japan) for 30 min at 37°C to remove residual DNA. RNA quality was verified using a 2100 Bioanalyzer (Agilent Technologies, Santa Clara, CA) and was also checked by RNase free agarose gel electrophoresis. The concentration of the total RNA was further quantified with a RNA NanoDrop (Thermo Fisher Scientific Inc., Waltham, MA, USA).

### cDNA library construction

Aliquots of 20 μg each of total RNA from the two different mung bean cultivars were separately processed for cNDA library construction. In both cases, a concentration of ≥ 400 ng/μl, OD260/280 = 1.8~2.2, RNA 28S:18S ≥ 1.0, and RNA Integrity Number (RIN) ≥ 7.0 was used for the preparation of a cDNA libraries. Poly-T oligonucleotide labeled magnetic beads (Illumina Inc., San Diego USA) was used to isolate poly (A) mRNA from the total RNA. Subsequently, the isolated mRNA was purified and fragmented into smaller pieces (200–700 nt) using divalent cations at 94°C for 5 min. First strand cDNA was synthesized with SuperScript II reverse transcriptase and random primers using the small fragment RNAs as templates. Second-strand cDNA synthesis was carried out using GEX second strand buffer, dNTPs, RNase H and DNA polymerase I. The cDNA fragments were further processed with end repair and phosphorylation using T4 DNA polymerase, Klenow DNA polymerase, and T4 polynucleotide kinase. The repaired cDNA fragments were 3’ adenylated using Klenow enzyme (Exo-) before end-ligating with Illumina paired-end adapters. The products from this ligation reaction were electrophoresed on a 2% (w/v) TAE-agarose gel and purified to select templates of different sizes for downstream enrichment. Only cDNA fragments of 200 bp (±25 bp) were excised from the gel and subjected to PCR. Thermocycling enrichment consisted of 15 cycles of PCR amplification performed using PCR primers PE1.0 and PE2.0 with Phusion DNA Polymerase.

### Illumina Sequencing, data filtering and *de novo* assembly

The new cDNA libraries of the two mung bean cultivars were sequenced with Illumina paired-end sequencing technology [23] and an Illumina Hiseq 2000 sequencer which automatically collected the data and generated FASTQ files (.fq) containing raw data for all the reads. The files for ZL1 and V6, based on cultivar, were submitted to the sequence read archive (SRA) database at Genbank (www.ncbi.nlm.nih.gov), where they were combined and given the accession number SRP043316. The raw data was stringently filtered for preliminary assembly. All reads with more than 10% of bases with a poor quality score (Q<20), or non-coding RNA (such as rRNA, tRNA and miRNA), as well as ambiguous sequences containing an excess of “N” nucleotide calls or adaptor contamination, were removed. We also discarded the reads that did not pass the Illumina failed-chastity filter according to the relation “failed-chastity ≤ 1”, with a chastity threshold of 0.6 on the first 25 cycles. After this, *de novo* transcriptome assembly was performed with the software Trinity [24] by uploading the high-quality reads onto a computer for further analysis to 1) reduce the graph complexity by resolving repetitive sequences shorter than the read length in the graph; 2) clip the short tips in the graphs with lengths less than 2K (58 bp); 3) filter the low-coverage links that appeared only once along with their related edges; 4) merge the detected bubbles into a single path if the sequences of the parallel paths had a difference of fewer than four base pairs with >90% identity. After all these steps, the connections on the simplified graphs were broken at any repeat boundaries. These bioinformatics processes resulted in sequences without redundancy that contained the least amount of “N” nucleotide calls un-extended on either end. Only these stringently compiled sequences were defined as unigenes.

### Unigene annotation and classification

The annotation of unigenes was performed using various bioinformatics procedures. The unigenes were aligned with BLASTX to four protein databases (NCBI non-redundant or Nr proteins, Swiss-Prot, Kyoto Encyclopedia of Genes and Genomes or KEGG and euKaryotic Ortholog Groups or KOG) and one nucleotide database (NCBI nucleotide or Nt sequences) with an E-value threshold of 1.0E-5 for all except KOG with a threshold of 1.0E-3 [25,26]. The proteins with highest sequence similarity were retrieved and annotated to each unigene. With nucleotide based annotation, Blast2GO [27] software was used to obtain GO annotation categories defined by molecular function, cellular component and biological process ontologies. The KOG database was used to predict possible functions while pathway assignments were determined with KEGG.

### EST-SSR search and primer design

The MIcroSAtellite (MISA) search engine (http://pgrc.ipk-gatersleben.de/misa) was employed for SSR mining and identification. The minimum numbers of repeats used for selecting the EST-SSRs were ten for mono-nucleotide based loci, six for di-nucleotide loci, five for tri-nucleotide loci and three for all larger repeat types (tetra- to hexa-nucleotide motifs). SSR marker primer pairs were designed based on sequences flanking the selected microsatellite loci using the software package Premier 5.0 (PREMIER Biosoft International, Palo Alto, CA) with targeted sizes of PCR products in the range between 100 to 300 bp.

### Marker validation and genomic DNA extraction

Validation of the EST-SSR markers was conducted with the 31 mung bean accessions mentioned previously. Genomic DNA was extracted from young leaves of these accessions using the Hexadecyl trimethyl ammonium Bromide (CTAB) extraction method [28]. DNA quality was evaluated on a 1.0% agarose gel electrophoresis. The working concentration of DNA was adjusted to 50 ng/ml for use in marker evaluations. Amplification was performed in 20 μl volume reactions containing 0.5 U of Taq DNA polymerase, 1 × PCR Buffer II, 1.5 mM MgCl2, 25 μM of dNTP, 0.4 μM of each primer, and 50 ng of genomic DNA. Microsatellite loci were amplified on a Heijingang Thermal Cycler (Eastwin, Beijing, China). PCR amplification was carried out with the following cycling conditions: one cycle of 4 min at 94°C, 30–35 cycles at 94°C for 30 s, 55–60°C for 30 s and 72°C for 30 s. The final extension was performed at 72°C for 10 min. The PCR products were analyzed by 8.0% non-denaturing PAGE (Polyacrylamide gel electrophoresis) using silver staining. Fragment sizes were estimated based on the 1 Kb size marker as a DNA ladder (Promega, Madison, WI, USA).

### Genetic similarity analysis

A distance tree was built based on a genetic similarity matrix for the 31 mung bean accessions and branch support was estimated with 10,000 bootstraps. The number of alleles (N_a_), observed heterozygosities (H_o_) and polymorphism information content (PIC) for each of the EST-SSR markers were calculated using the software POPGEN 1.32 [29]. The cluster analysis of genotypes was carried out based on Nei’s unbiased measures of genetic distance by using the unweighted pair-group method with arithmetic average (UPGMA) and coefficients of genetic similarity for the mung bean accessions calculated using the same program [29].

## Results

### Sequencing and *de novo* assembly of Illumina paired-end reads

A total of 52.7 and 51.7 million paired-end raw reads were generated in Illumina next generation sequencing runs for the ZL1 and V6 varieties, respectively. After removal of the low quality reads, 51.9 and 50.9 million clean reads remained, with GC content of 43.1% and 44.8% in ZL1 and V6, respectively. In terms of sequence quality, ZL1 and V6 had 98.4% and 98.2% of Q20 or above bases and 94.5% and 93.9% of Q30 or above bases, respectively.

**Fig 1:**
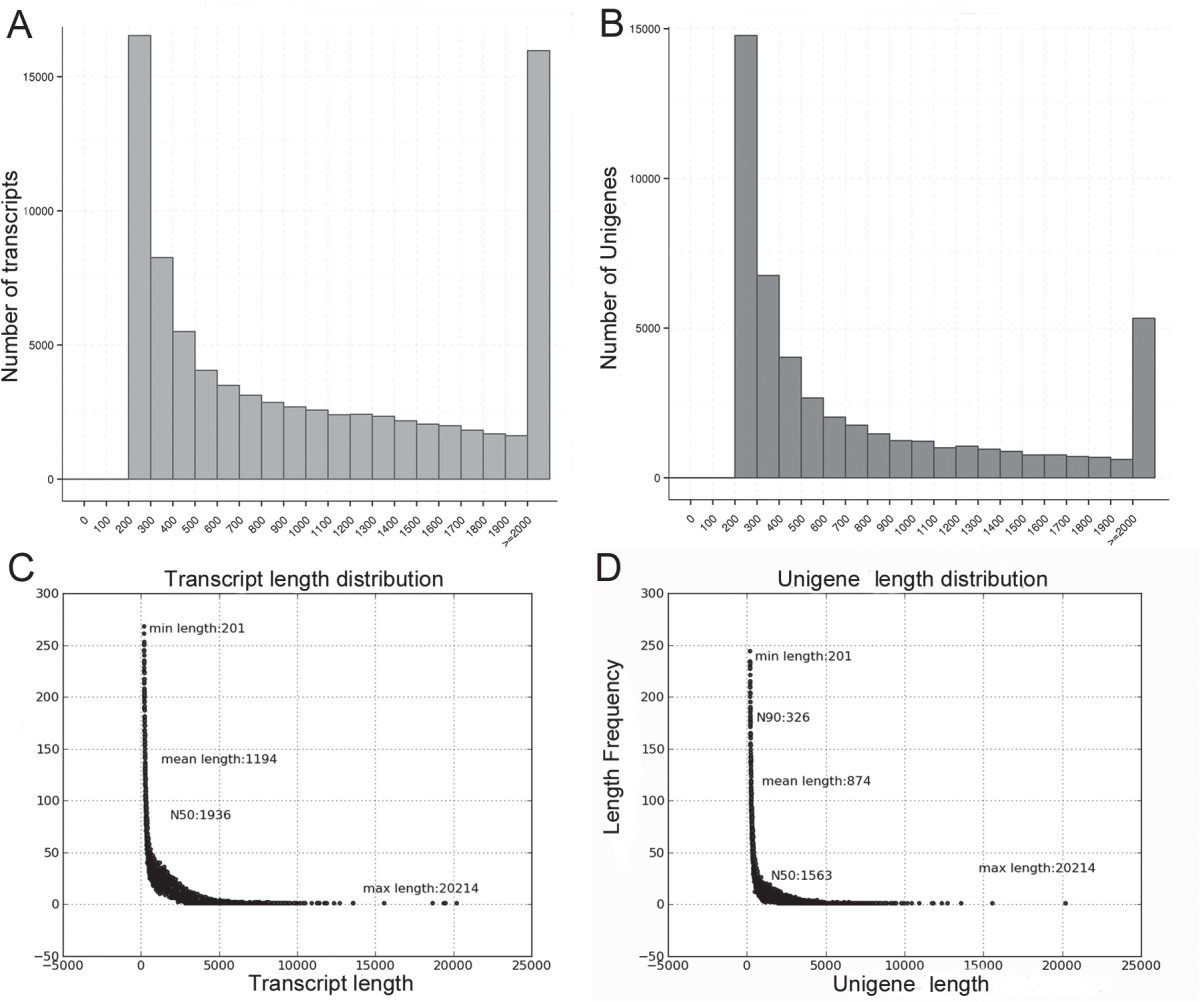
Length distribution of assembled transcripts and unigenes. **A.** Size distribution of transcripts. B. Size distribution of Unigenes. C. Length distribution of transcripts. D. Length distribution of Unigenes.

The combined sequence length of the Illumina reads was 10.3 Gb and could be assembled *de novo* into 48,693 unigenes and 83,542 individual transcripts. The average length of the assembled transcripts was 1,194 bp (N50 = 1,936 bp), which was longer than the average length of the assembled unigenes (874 bp, N50 = 1,563 bp). The range in length of the assembled unigenes was from 200 bp to 20,214 bp. A total of 25,590 unigenes (52.6%) were short, with lengths no longer than 500 bp. The next two size classifications of unigenes were of similar frequency with 9,141 unigenes (18.8%) having 501 to 1,000 bp in length, and 8,643 unigenes (17.8%) with lengths ranging from 1,000 to 2,000 bp. Finally 5,319 unigenes (10.9%) were longer than 2,000 bp (Fig 1).

### Sequence annotation

For validation and annotation of the sequence assembly contigs and unique singletons, all unigenes were searched against the five databases as described earlier. A total of 48,693 unigenes provide a significant BLAST result, with 25,820 (53.0%) showing significant similarity to known proteins in the Nr sequence database, with 18,316 from Swiss-Prot (37.6%) and 17,652 from PFAM (36.3%), only 4,064 unigenes were annotated in all databases but 28,613 could be annotated in at least one while the rest (20,080 unigenes) were not annotated to the existing databases.

**Fig 2:**
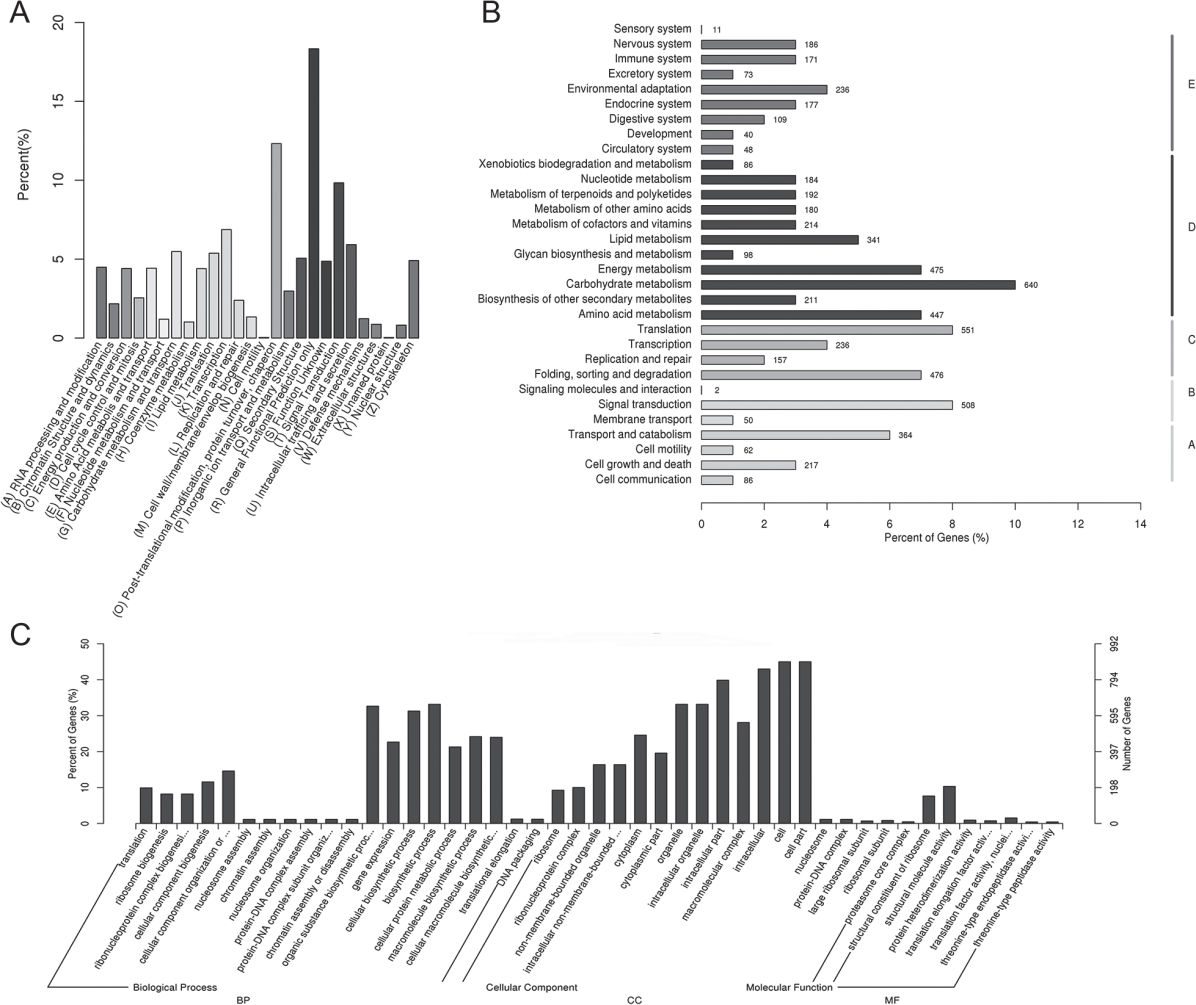
Classification of assembled unigenes. A. EuKaryotic Ortholog Groups (KOG) classification of assembled unigenes. B. Gene ontology (GO) classification of assembled unigenes. C. Kyoto Encyclopedia of Genes and Genomes (KEGG) classification of assembled unigenes.

Assembled unigenes were classified in various ways (Fig 2). Based on Nr annotation, 19,242 unigenes (39.5%) were assigned gene ontology (GO) terms (Fig 2A). The sequences that belonged to the biological process, cellular component, and molecular function clusters were categorized into 55 functional groups. Binding (11,440, 59.5%), cellular processes (11,392, 59.2%), metabolic processes (11,049, 57.4%), catalytic activity (9,595, 49.9%) and cell part (6,524, 33.9%) were the dominant five groups respectively, however, only 1 unigene each was assigned to metallo-chaperone activity and symplast localization.

After functional prediction and classification, 10,918 unigenes (22.4%) could be classified by hits with the KOG database (Fig 2B). The KOG-annotated putative proteins were functionally classified into 26 molecular families. General function only (2,001, 19.3%), post-translational modification, protein turn over and chaperon activity (1,345, 13.0%), signal transduction (1,074, 10.4%), transcription (750, 7.2%) and intracellular trafficking (646, 6.2%) were the dominant five groups, whereas only 4 unigenes each were assigned to cell motility and un-named protein groupings. To further analyze the transcriptome of mung bean, all the unigenes were analyzed in the KEGG database (Fig 2C), where a total of 6,585 unigenes had significant matches and were assigned to 5 main categories in 244 pathways. Among these positive KEGG hit unigenes, metabolic pathways contained 3,068 unigenes, followed by genetic information processing (1,420, 21.6%), organismal systems (1,051, 16.0%), cellular processes (729, 11.1%) and environmental information processing (560, 8.5%).

### Frequency and distribution of different types of EST-SSR markers

Of the 48,693 unigenes found in the current study, 10,053 (20.6%) contained one or more SSR sequences. Of these, 2,303 (4.9%) contained at least two separate SSR sequences and 978 (2.1%) contained compound SSRs of different motifs. The proportion of EST-SSR was not evenly distributed among EST-SSR unit sizes or groups. Mono-nucleotide motifs were the most abundant (4,751, 36.2%) with tetra- (2,813, 21.4%), tri- (1,915, 14.6%), di- (1,809, 13.8%), penta-nucleotide (969, 7.4%) and hexa- (877, 6.7%) nucleotide motif repeats being the next most common in consecutive order (Table 1).

**Table 1:** Summary of the number of repeat units in mung bean EST-SSR loci.

**SSR motif length**	**Repeat unit number**										
	**3**	**4**	**5**	**6**	**7**	**8**	**9**	**10**	**>10**	**Total**	**%**
Mono-	-	-	-	-	-	-	-	1,978	2,773	4,751	36.2
Di-	-	-	-	638	350	289	193	217	122	1,809	13.8
Tri-	-	-	1,048	510	330	24	1	0	2	1,915	14.6
Tetra-	2,443	283	72	14	0	1	0	0	0	2,813	21.4
Penta-	828	131	8	1	0	0	0	0	1	969	7.4
Hexa-	742	123	3	2	2	2	1	1	1	877	6.7
Total	4,013	537	1,131	1,165	682	316	195	2,196	2,899	13,134	
%	30.6	4.1	8.6	8.9	5.2	2.4	1.5	16.7	22.1		

The number of SSR repeats per locus ranged from 3 to 23, and SSRs with three repeats were the most abundant, followed by those with ten, six and five random repeats. Motifs that showed more than 16 repeats were rare, with a frequency of only 4.4%. Among mono-nucleotide repeats, the (A/T)_n_ repeats were far more abundant (99.7%) compared to the (G/C)_n_ repeats. The six other main motif types were the (AG/CT)_n_ di-nucleotide repeat (30.8%), the (GAA/TTC)_n_ tri-nucleotide repeat (12.6%), the (AAAT/ATTT)_n_ tetra-nucleotide repeat (6.8%), the (AAAAT/ATTTT)_n_ penta-nucleotide repeat (6.2%), and then the (AAAAAT/ATTTTT)_n_ hexa-nucleotide repeat (1.9%), consecutively (S2 Dataset).

### Development of polymorphic EST-SSR markers in mung bean

A total of 13,134 *in silico* EST-SSR markers could be developed form the 10,053 SSR containing sequences using Primer3 (S3 Dataset). A subset of 200 markers was randomly chosen from these loci to validate EST-SSR marker usefulness in monitoring polymorphisms for eight mung bean accessions (S4 Dataset). Of the markers tested, 129 primer pairs (65.0%) produced clear PCR amplicons of the expected sizes, 36 markers amplified non-specific products, and 35 did not amplify any clear DNA bands. Of the successfully amplifying EST-SSR markers, 66 (or 51.2%) were polymorphic and consisted of 4 mono-, 2 di-, 33 tri-, 6 tetra-, 3 penta- and 18 hexa motif based marker (Table 2) while the other 97 markers were monomorphic. An average of 3.0, 2.5, 2.2, 2.2, 2.0 and 2.6 alleles were generated for the mono-, di-, tri-, tetra-, penta- and hexa motif markers, respectively.

**Table 2:** The evaluation of microsatellite markers for different repeat classes.

**Class**	**Tested markers (%)**	**Scorable markers (%)**	**Polymorphic markers (%)**	**Mean of alleles per locus** ± **SD** ^1^	**Marker PIC value** ± **SD** ^1^
Mono-	7 (3.5)	5	4 (80.0)	3.0±0.50	0.290 ±0.066
Di-	8 (4.0)	7	2 (28.6)	2.5±0.71	0.363±0.016
Tri-	72 (36.0)	60	33 (53.3)	2.2±0.42	0.337±0.117
Tetra-	7 (3.5)	6	6 (100.0)	2.2±0.41	0.320±0.053
Penta-	4 (2.0)	4	3 (75.0)	2.0±0.00	0.359±0.024
Hexa-	102 (51.0)	81	18 (22.2)	2.6+0.86	0.372+0.091
Total (average)	200	163	66	(2.3)	(0.344)

^1^Standard Deviation.

### Gene functions of the unigene sequences containing polymorphic EST-SSRs

To determine the possible functions of the 66 validated EST-SSRs, they were subjected to BLASTn analysis with a non-redundant database of legume sequences. The results showed that most of the sequences were similar to known or hypothetical protein-encoding genes from common bean (*Phaseolus vulgaris* L.) and soybean (*Glycine max* L.) with a lesser proportion homologous to cowpea (*Vigna unguiculata* L. [Walp]) genes (S5 Dataset). Among the positive hits were genes for auxin efflux carrier component, dof zinc finger, F-box, gibberellin receptor, helicase, mitogen-activated and leucine-rich repeat extensin-like proteins as examples.

### Phylogenetic analysis of the cultivated mung bean accessions

The 66 polymorphic EST-SSR markers developed in this study were used to assess the genetic diversity of 31 mung bean accessions from the complete geographic distribution of the crop for which a total of 154 alleles were detected and scored. The number of alleles per marker ranged from 2 to 5. Effective number of alleles per locus (N_e_) varied from 1.074 (for marker MB64504) to 3.014 (MB27164) averaging 1.810, expected heterozygosity (H_e_) ranged from 0.070 (MB64504) to 0.675 (MB9309) averaging 0.429, observed heterozygosity (H_o_) varied from 0 (MB21076) to 0.897 (MB27164) averaging 0.100. Shannon's Information index (I) values ranged from 0.154 (MB64504) to 1.259 (MB27164) averaging 0.649 and PIC values ranged from 0.067 (MB17985) to 0.613 (MB25181) averaging 0.344 (Table 3).

**Table 3:** Informativeness of EST-SSR loci following amplification from 31 geographically diverse accessions of mung bean.

**Locus**	**Na** ^1^	**Ne** ^2^	**He** ^3^	**Ho** ^4^	**I** ^5^	**PIC** ^6^
MB10859	3	1.705	0.420	0.161	0.687	0.351
MB24080	2	1.385	0.283	0.111	0.451	0.239
MB19587	2	1.788	0.448	0.035	0.633	0.340
MB19823	2	1.355	0.267	0.035	0.432	0.374
MB22860	2	1.998	0.508	0.138	0.693	0.282
MB10675	2	1.839	0.465	0.037	0.649	0.372
MB11384	2	1.897	0.481	0.033	0.666	0.565
MB29365	2	1.516	0.348	0.087	0.524	0.333
MB9044	3	1.615	0.389	0.080	0.659	0.587
MB9309	3	2.955	0.675	0.120	1.091	0.382
MB16266	2	1.174	0.151	0.161	0.280	0.137
MB23088	2	1.508	0.343	0.071	0.520	0.332
MB16558	2	1.857	0.475	0.167	0.654	0.280
MB14327	2	1.874	0.475	0.148	0.659	0.299
MB21076	2	1.981	0.503	0.000	0.689	0.355
MB17669	2	1.708	0.422	0.103	0.605	0.325
MB14798	3	1.515	0.346	0.069	0.603	0.375
MB15159	2	1.938	0.493	0.036	0.677	0.358
MB15469	2	1.934	0.492	0.074	0.676	0.373
MB31003	2	1.301	0.235	0.067	0.393	0.329
MB33094	2	1.454	0.317	0.065	0.491	0.346
MB21347	3	2.822	0.656	0.032	1.069	0.470
MB19157	2	1.903	0.482	0.065	0.667	0.332
MB29460	2	1.991	0.506	0.172	0.691	0.301
MB25181	2	1.753	0.439	0.208	0.621	0.613
MB55107	2	1.990	0.507	0.071	0.691	0.330
MB9543	2	1.800	0.452	0.000	0.637	0.366
MB52717	2	1.998	0.508	0.367	0.693	0.448
MB26622	2	1.251	0.204	0.097	0.353	0.352
MB26637	2	1.432	0.308	0.074	0.479	0.204
MB26838	2	1.690	0.416	0.071	0.598	0.263
MB22833	2	1.212	0.178	0.194	0.318	0.215
MB19617	2	1.949	0.495	0.194	0.680	0.352
MB64504	2	1.074	0.070	0.000	0.154	0.361
MB27164	5	3.014	0.680	0.897	1.259	0.383
MB15686	2	1.969	0.503	0.125	0.685	0.573
MB56315	2	1.800	0.452	0.067	0.637	0.362
MB22940	2	1.338	0.257	0.000	0.420	0.374
MB14180	3	2.839	0.658	0.065	1.070	0.223
MB2421	3	1.595	0.379	0.226	0.681	0.375
MB27639	2	1.578	0.373	0.069	0.553	0.351
MB16610	2	1.835	0.463	0.100	0.647	0.368
MB27721	3	1.484	0.332	0.033	0.558	0.337
MB11596	3	1.811	0.457	0.000	0.778	0.374
MB25166	2	1.415	0.299	0.000	0.469	0.283
MB37870	2	1.991	0.506	0.172	0.691	0.340
MB21522	2	1.976	0.503	0.148	0.687	0.344
MB51985	2	1.724	0.427	0.000	0.611	0.228
MB13673	3	2.776	0.652	0.115	1.057	0.346
MB34120	3	1.770	0.442	0.033	0.751	0.374
MB15445	3	1.546	0.359	0.097	0.658	0.180
MB79303	2	1.800	0.452	0.133	0.637	0.256
MB24478	4	2.125	0.540	0.000	0.956	0.547
MB8236	2	1.724	0.427	0.133	0.611	0.325
MB22067	2	1.715	0.425	0.074	0.608	0.160
MB11659	3	2.096	0.532	0.167	0.866	0.397
MB29754	2	1.324	0.249	0.000	0.410	0.368
MB17985	2	1.897	0.481	0.033	0.666	0.067
MB25181	3	1.867	0.472	0.000	0.745	0.367
MB19286	2	1.342	0.259	0.033	0.423	0.368
MB24843	2	2.000	0.512	0.143	0.693	0.250
MB22568	2	1.830	0.464	0.087	0.646	0.346
MB25254	2	1.942	0.494	0.138	0.678	0.221
MB15212	2	1.766	0.444	0.182	0.626	0.574
MB25564	3	2.670	0.636	0.000	1.031	0.334
MB10515	2	1.734	0.433	0.000	0.615	0.361

^1^The number of observed alleles.

^2^The number of effective number of alleles.

^3^The number of expected heterozygosity.

^4^The number of observed heterozygosity.

^5^Shannon's Information index (Lewontin, 1972).

^6^Polymorphic information content.

**Fig 3:**
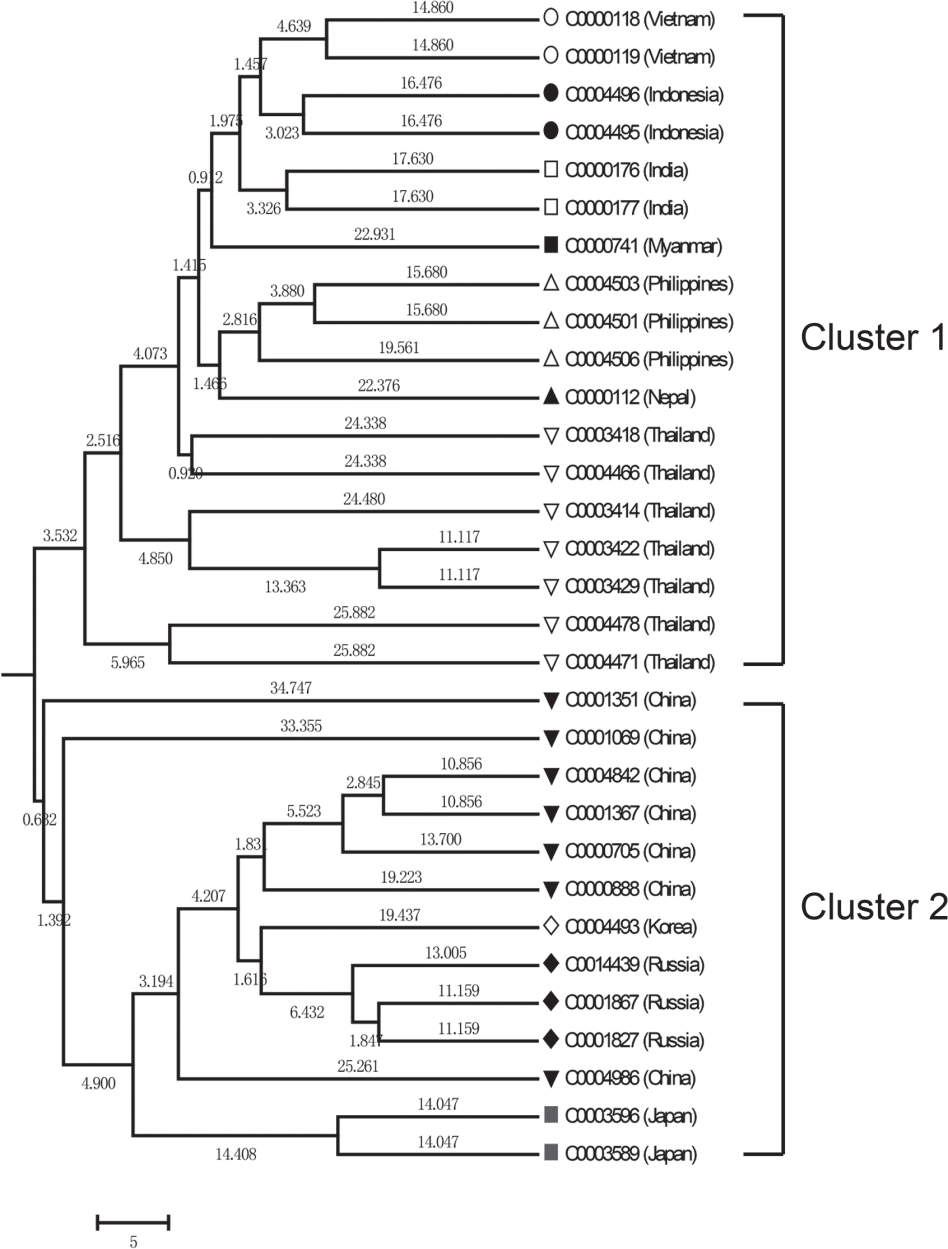
UPGMA dendrogram of 31 genotypes of mung bean.

Phylogenetic relationships between the accessions grouped the 31 accessions into two main clusters in a dendogram (Fig 3). Cluster 1 was comprised of accessions from Southeast and South Asian countries such as Thailand, Vietnam, Philippines, Indonesia, Myanmar, Nepal and India. Cluster 2 was comprised of accessions from East Asian and Northeast Asian countries such as China, Japan, Korea and Russia. Results indicated that geographical distances between collection sites for the accessions were associated with Nei’s genetic distances between accessions.

## Discussion

Transcriptome sequencing and *de novo* assembly has proven to be an important tool for gene discovery in many organisms and an effective method for molecular marker development [30,31]. Our results also proved that the short reads from Illumina paired-end sequencing of mung bean cDNAs can be easily assembled and used for transcriptome analysis, marker development and gene identification even without a reference genome for the crop. The marker validation confirmed previous evaluations of SSRs in common bean [8,32] and other legume crops, where EST-SSR markers detect moderate polymorphism.

Our work complements previous analysis with 454 sequencing of two mung bean cDNA libraries which resulted in the discovery of 1,630 and 1,334 EST-SSR primer pairs from the leaves of Jangan and Sunhwa varieties, respectively [16]. Here we concentrated on the use of Illumina sequencing to develop a larger total number of *in silico* EST-SSR markers to increase the number of SSRs available for mung bean. We found that the EST-SSR marker validation rate was similar to the success rate of SSR development in a previous study in mung bean [33] but slightly lower than when using sequenced BAC end sequences or small-insert genomic libraries in common bean [34,35]. Comparing these legumes, the polymorphism ratio of EST-SSR markers in mung bean was slightly higher than for EST-SSR in common bean [18].

In terms of the types of motifs found in SSR loci other than the mono- and large sized repeats, we found similar results as in previous work with plant microsatellites [33]. For example, the proportions of di- and tri-nucleotide repeats were quite close (13.8% *versus* 14.6%) as was found in previous results [30] The relative abundance of di- and tri-nucleotide repeats in ESTs sequences has been observed in many other legumes including common bean [8], cowpea [36] and chickpea [37]. The most common tri-nucleotide repeats found in the mung bean varieties studied here were GAA/TTC followed by TCT/AGA and CTT/AAG, which are similar with previous reports in mung bean [17] and common bean [8,18], possibly indicating a shared origin among the *Phaseoleae* tribe. As in common bean, AG/CT motif was the most abundant repeat motif (30.8%), followed by AT/TA (28.5%) contrasting slightly with other legumes outside the *Phaseoleae* tribe [36–38] but similar to estimates in common bean [8].

A larger number of repeat units were generally correlated with greater allelic variability for an SSR locus. Therefore, the shorter motif loci such as those with mono- and di-nucleotides repeats usually had to possess more repeats to be of equivalent polymorphism to longer motif repeats such as those with tri-nucleotide motifs. Previous studies in legumes have mainly focused on di-, tri-, and tetra-nucleotide SSRs [18], whereas mono-nucleotide SSRs perhaps have not drawn enough attention for marker development. We found that mono-nucleotide SSRs had higher polymorphism rates than previously thought, followed by tetra-nucleotide SSRs, justifying their inclusion in future SSR evaluations.

To determine the level of polymorphism of our new EST-SSR markers, we validated 200 loci, of which 129 markers (65.0%) produced successful amplicons, which is in between previously reported success rates of 21.0% [15] and 78.0% [17] in mung bean. The failure of 35 primer pairs to generate amplicons may be due to long intervening introns which would not allow successful genomic amplification of markers based on transcribed mRNA based sequences. Alternatively the location of primers across splice sites or regions of poor sequence quality could explain non-amplification.

Despite these issues, about two thirds of the EST-SSR markers were successful, suggesting that the transcriptome sequencing was accurate and the assembled unigenes were of high quality. In terms of allele detection, only half of the successfully amplified SSRs produced more than one allele and most had no more than 4 alleles, which was in agreement with a previous study [39] for mung bean. PIC values in this study were in line with previously reported values for mung bean SSRs [17,40]. They were also similar to EST-SSRs from adzuki bean which can amplify products in mung bean [41]. One advantage of EST-SSR markers, is that they may detect valuable genetic diversity possibly associated with traits of interest for breeding because of their location in genes.

In summary, the accomplishments of our study were 1) the detection of a large number of unigenes for mung bean and 2) the discovery of over 10,000 SSR containing sequences in the transcriptome of the crop. We observed that the number and lengths of unigenes in mung bean compared favorably to previous analyses with the generation here of approximately 25 million paired-end reads for the transcriptome which assembled into over 48 thousand unigenes with an average length of over 850 bp. Sanger sequencing of cDNAs do not efficiently produce this number of unigenes or sufficient overall contig lengths because of a limitation in the depth of sequencing, even when full-length cDNA libraries are used [31,42,43]. The use of transcriptomic data for *in silico* microsatellite development was shown to be promising and we were able to increase the number of possible EST-SSRs tenfold compared to previous studies [33]. The newly developed SSR sequences and EST-SSR markers we made will be important resources for basic research and together with SNP resources can significantly enhance the ability to find closely linked markers for traits of interest in the molecular breeding of mung bean.

## Supporting Information

S1 Dataset Germplasm accessions used in this study of mung bean. (DOC)

S2 Dataset Frequencies of different repeat motifs in EST-SSRs from mung bean. (DOC)

S3 Dataset Characteristics of 13,134 mung bean EST-SSR markers in this study. (XLS)

S4 Dataset Primer sequences of a total of 200 SSR markers for validation. (XLS)

S5 Dataset The putative proteins identified by BLASTX of 66 unigene sequences containing polymorphic EST-SSRs. (DOC)
