# Supplementary material for: Transcriptome Sequencing of Mung Bean (Vigna radiate L.) Genes and the Identification of EST-SSR Markers
Source: PLoS One. 2015 Apr 1;10(4):e0120273. doi: 10.1371/journal.pone.0120273 (PMC4382333; doi:10.1371/journal.pone.0120273)
Supplement: S1 Dataset — (DOC) [file pone.0120273.s001.doc]

**Table S1. Germplasm accessions used in this study of mungbean.**

| Serial No. | Accession number | Country of origin | Cultivar name |
| --- | --- | --- | --- |
| 1 | C0000112 | Nepal | YJ013356 |
| 2 | C0000118 | Vietnam | VN118 |
| 3 | C0000119 | Vietnam | VN119 |
| 4 | C0000176 | India | TAR-3 |
| 5 | C0000177 | India | NL-17 |
| 6 | C0000705 | China | Xiaoyinggedou |
| 7 | C0000741 | Myanmar | MY1 |
| 8 | C0000888 | China | Lvdou2 |
| 9 | C0001069 | China | Xiaomingxing |
| 10 | C0001351 | China | Zhonglv5 |
| 11 | C0001367 | China | Zhonglv12 |
| 12 | C0001827 | Russia | RS1827 |
| 13 | C0001867 | Russia | RS1867 |
| 14 | C0003414 | Thailand | VC2778A |
| 15 | C0003418 | Thailand | V1381 |
| 16 | C0003422 | Thailand | VC2750A |
| 17 | C0003429 | Thailand | VC3301A |
| 18 | C0003586 | Japan | JP362302 |
| 19 | C0003589 | Japan | SES-55 |
| 20 | C0004439 | Russia | RS14439 |
| 21 | C0004466 | Thailand | VC2917A |
| 22 | C0004471 | China | VC3738A |
| 23 | C0004478 | China | VC3890 |
| 24 | c0004493 | Korea | KR4493 |
| 25 | C0004495 | Indonesia | IN4495 |
| 26 | C0004496 | Indonesia | IN4496 |
| 27 | C0004501 | Philippines | PH4501 |
| 28 | C0004503 | Philippines | PH4503 |
| 29 | C0004506 | Philippines | PH4506 |
| 30 | C0004842 | China | Yinggelv |
| 31 | C0004986 | China | Lvdou |

Note: these accession were from National Center for Crop Germplasm Resources Preservation of China, accession 3, 5, 12, 15, 21, 22, 28 and 30 were used for screening of EST-SSR.
