# Supplementary material for: Transcriptome Sequencing of Mung Bean (Vigna radiate L.) Genes and the Identification of EST-SSR Markers
Source: PLoS One. 2015 Apr 1;10(4):e0120273. doi: 10.1371/journal.pone.0120273 (PMC4382333; doi:10.1371/journal.pone.0120273)
Supplement: S2 Dataset — (DOC) [file pone.0120273.s002.doc]

**Table S2. Frequencies of different repeat motifs in EST-SSRs from mung bean.**

| **Repeats motif** |  | **Number of motif repeats** | | | | | | | | | | |
| --- | --- | --- | --- | --- | --- | --- | --- | --- | --- | --- | --- | --- |
|  | **3** | **4** | **5** | **6** | **7** | **8** | **9** | **10** | **11** | **12** | **>12** | **Total** |
| A/T | - | - | - | - | - | - | - | 1973 | 959 | 494 | 1311 | 4737 |
| AG/CT | - | - | - | 191 | 101 | 101 | 69 | 63 | 31 | 2 | - | 558 |
| TA/AT | - | - | - | 170 | 86 | 73 | 59 | 77 | 48 | 1 | 1 | 515 |
| TC/GA | - | - | - | 164 | 117 | 80 | 54 | 69 | 33 | - | - | 497 |
| GAA/TTC | - | - | 116 | 80 | 42 | 3 | - | - | - | - | - | 241 |
| TCT/AGA | - | - | 98 | 55 | 37 | 3 | - | - | - | - | - | 194 |
| CTT/AAG | - | - | 75 | 38 | 32 | 3 | - | - | - | - | - | 148 |
| AAAT/ATTT | 167 | 19 | 5 | - | - | - | - | - | - | - | - | 191 |
| TTTA/TAAA | 149 | 20 | 7 | - | - | - | - | - | - | - | - | 176 |
| TTTC/GAAA | 129 | 14 | 10 |  | - | - | - | - | - | - | - | 153 |
| AAGA/TCTT | 128 | 8 | 4 | - | - | 1 | - | - | - | - | - | 141 |
| AAAG/CTTT | 113 | 14 | 1 | 2 | - | - | - | - | - | - | - | 130 |
| AAAAT/ATTTT | 52 | 7 | - | - | - | - | - | - | 1 | - | - | 60 |
| AAAAG/CTTTT | 30 | 4 | - | - | - | - | - | - | - | - | - | 34 |
| TTTTC/GAAAA | 26 | 4 | - | 1 | - | - | - | - | - | - | - | 31 |
| TCTTC/GAAGA | 20 | 10 | - | - | - | - | - | - | - | - | - | 30 |
| TTTTA/TAAAA | 24 | 1 | - | - | - | - | - | - | - | - | - | 25 |
| AGAGA/TCTCT | 17 | 6 | - | - | - | - | - | - | - | - | - | 23 |
| AATAA/TTATT | 19 | 3 | - | - | - | - | - | - | - | - | - | 22 |
| AAAAAT/ATTTTT | 16 | 1 | - | - | - | - | - | - | - | - | - | 17 |
| AAAAAG/CTTTTT | 9 | 2 | - | - | - | - | - | - | - | - | - | 11 |
| AGAGAA/TTCTCT | 10 | 1 | - | - | - | - | - | - | - | - | - | 11 |
