# Supplementary material for: Transcriptome Sequencing of Mung Bean (Vigna radiate L.) Genes and the Identification of EST-SSR Markers
Source: PLoS One. 2015 Apr 1;10(4):e0120273. doi: 10.1371/journal.pone.0120273 (PMC4382333; doi:10.1371/journal.pone.0120273)
Supplement: S5 Dataset — (DOC) [file pone.0120273.s005.doc]

**Table S5. The putative proteins identified by BLASTX of 66 unigene sequences containing polymorphic EST-SSRs.**

| **Primer Name** | **Organism**  **(Phaseolus vulgraris)** | **GenBank (Accession No.)** | **Organism**  **(Glycine max)** | **GenBank (Accession No.)** |
| --- | --- | --- | --- | --- |
| MB10859 | Hypothetical protein (PHAVU_001G080700g) mRNA | XM_007161508.1 | Uncharacterized LOC100781733 (LOC100781733) | XM_006606351.1 |
| MB24080 | Hypothetical protein (PHAVU_005G027400g) mRNA | XM_007148880.1 | F-box protein At3g54460-like (LOC100779718) | XM_006578429.1 |
| MB19587 | Hypothetical protein (PHAVU_005G133500g) mRNA | XM_007150120.1 | Helicase protein MOM1-like (LOC100810773) | XM_006594911.1 |
| MB19823 | Hypothetical protein (PHAVU_010G163000g) mRNA | XM_007135782.1 | Mitogen-activated protein kinase kinase 4-like (LOC100818488), | XM_003531723.2 |
| MB22860 | Hypothetical protein (PHAVU_009G113000g) mRNA | XM_007137203.1 | Putative lipase ROG1-like (LOC100784218) | XM_003523711.2 |
| MB10675 | No hit | – | Glutathione S-transferase GST 11 (LOC548029) | NM_001250741.2 |
| MB11384 | Hypothetical protein (PHAVU_006G103200g) mRNA | XM_007147126.1 | Uncharacterized LOC100795835 (LOC100795835) | XM_003521703.2 |
| MB29365 | Hypothetical protein (PHAVU_003G101200g) mRNA | XM_007154168.1 | CASP-like protein 5 (LOC100500041) | NM_001249080.2 |
| MB9044 | Hypothetical protein (PHAVU_001G146300g) mRNA | XM_007162308.1 | Gibberellin receptor GID1B-like (LOC100788435) | XM_006576817.1 |
| MB9309 | Hypothetical protein (PHAVU_006G200900g) mRNA, | XM_007148289.1 | Guanine nucleotide-binding protein-like 3 homolog (LOC100784633) | XM_003547056.2 |
| MB16266 | Hypothetical protein (PHAVU_002G307200g) mRNA | XM_007160209.1 | Leucine-rich repeat extensin-like protein 6-like (LOC100820611) | NM_001254000.2 |
| MB23088 | Hypothetical protein (PHAVU_001G121100g) mRNA | XM_007162008.1 | Auxin efflux carrier component 1-like (LOC100802041) | NM_001289386.1 |
| MB16558 | Hypothetical protein (PHAVU_002G142900g) mRNA | XM_007158259.1 | Probablebeta-D-xylosidase 6-like (LOC100783609) | XM_003534213.2 |
| MB14327 | No hit | – | No hit | – |
| MB21076 | Hypothetical protein (PHAVU_009G125900g) mRNA | XM_007137361.1 | NAC domain protein (NAC7) mRNA | EU661909.1 |
| MB17669 | Hypothetical protein (PHAVU_001G212100g) mRNA | XM_007163105.1 | Probablebeta-D-xylosidase 2-like (LOC100789316) | XM_003520701.2 |
| MB14798 | Hypothetical protein (PHAVU_005G119400g) mRNA | XM_007149961.1 | Uncharacterized LOC102669063 (LOC102669063) | XM_006594861.1 |
| MB15159 | Hypothetical protein (PHAVU_008G004600g) mRNA | XM_007139075.1 | Probablecarboxylesterase 17-like (LOC100804774) | XM_003530862.2 |
| MB15469 | Hypothetical protein (PHAVU_010G043000g) mRNA | XM_007134319.1 | Uncharacterized LOC100499981 (LOC100499981) | NM_001248221.1 |
| MB31003 | Hypothetical protein (PHAVU_005G023800g) mRNA | XM_007148842.1 | No hit | – |
| MB33094 | Hypothetical protein (PHAVU_004G174100g) mRNA | XM_007152892.1 | Leucine-rich repeat extensin-like protein 4-like (LOC100775544) | XM_006572780.1 |
| MB21347 | Hypothetical protein (PHAVU_003G006800g) mRNA, | XM_007153038.1 | Xyloglucan galactosyltransferase KATAMARI1 homolog (LOC100793938) | XM_003556564.2 |
| MB19157 | Hypothetical protein (PHAVU_002G333300g) mRNA | XM_007160512.1 | No hit | – |
| MB29460 | Hypothetical protein (PHAVU_001G238500g) mRNA, | XM_007163427.1 | Rop guanine nucleotide exchange factor 5-like (LOC100780972) | XM_003538385.2 |
| MB25181 | Hypothetical protein (PHAVU_005G180200g) mRNA | XM_007150721.1 | Mediator of RNA polymerase II transcription subunit 15a-like (LOC100783762) | XM_003547575.2 |
| MB55107 | Hypothetical protein (PHAVU_007G255500g) mRNA | XM_007145571.1 | Protein TRANSPARENT TESTA 1-like (LOC100783088) | XM_003519016.2 |
| MB9543 | Hypothetical protein (PHAVU_004G054500g) mRNA | XM_007151468.1 | Uncharacterized LOC100790222 (LOC100790222) | XM_003553800.2 |
| MB52717 | No hit | – | No hit | – |
| MB26622 | Hypothetical protein (PHAVU_009G252700g) mRNA | XM_007138896.1 | Protein DEHYDRATION-INDUCED 19 homolog 4-like (LOC102669504) | XM_006605785.1 |
| MB26637 | Hypothetical protein (PHAVU_010G149900g) mRNA | XM_007135624.1 | Splicing factor, arginine/serine-rich 19-like (LOC100808311) | XM_006605785.1 |
| MB26838 | Hypothetical protein (PHAVU_007G001400g) mRNA | XM_007142540.1 | Phosphoglucan, water dikinase, chloroplastic-like (LOC100783273) | XM_006589738.1 |
| MB22833 | Hypothetical protein (PHAVU_008G263700g) mRNA | XM_007142173.1 | Probableanion transporter 4, chloroplastic-like (LOC100789297) | XM_003519194.2 |
| MB19617 | Hypothetical protein (PHAVU_001G020400g) mRNA | XM_007160771.1 | 26S proteasome non-ATPase regulatory subunit 14 homolog (LOC100778174) | XM_003544406.2 |
| MB64504 | Hypothetical protein (PHAVU_001G080800g) mRNA | XM_007161509.1 | Dof zinc finger protein DOF4.6-like (LOC100782268) | XM_006606352.1 |
| MB27164 | mitochondrial import inner membrane translocase subunit tim17 Mrna | KF033606.1 | Mitochondrial import inner membrane translocase subunit TIM17-2-like (LOC100786954) | XM_003516740.2 |
| MB15686 | Hypothetical protein (PHAVU_008G180200g) mRNA | XM_007141189.1 | Glucose-6-phosphate isomerase 1, chloroplastic-like (LOC100791285) | XM_003522706.2 |
| MB56315 | Hypothetical protein (PHAVU_009G033900g) mRNA | XM_007136227.1 | RING-H2 finger protein ATL11-like (LOC100805815) | XM_003527845.2 |
| MB22940 | Hypothetical protein (PHAVU_009G071000g) mRNA | XM_007136687.1 | Activating signal cointegrator 1 complex subunit 2-like (LOC100778129) | XM_003523568.2 |
| MB14180 | Hypothetical protein (PHAVU_009G159400g) mRNA | XM_007137770.1 | Protein FRIGIDA-like (LOC100801324) | XR_414068.1 |
| MB2421 | Hypothetical protein (PHAVU_001G117800g) mRNA | XM_007161969.1 | Phosphatidylinositol 4-kinase gamma 8-like (LOC100809677) | XM_006604248.1 |
| MB27639 | Hypothetical protein (PHAVU_004G171000g) mRNA | XM_007152852.1 | Uncharacterized LOC100787849 (LOC100787849) | XR_136263.2 |
| MB16610 | WD repeat domain phosphoinositide-interacting protein 3 mRNA | KF033551.1 | Autophagy-related protein 18a-like (LOC100800215) | XM_003536023.2 |
| MB27721 | Hypothetical protein (PHAVU_005G130300g) mRNA | XM_007150079.1 | Zinc finger protein ZAT5-like (LOC100788646) | XM_006592658.1 |
| MB11596 | Hypothetical protein (PHAVU_006G213400g) mRNA | XM_007148432.1 | Pentatricopeptide repeat-containing protein At3g49710-like (LOC100788251) | XM_003551906.2 |
| MB25166 | No hit | – | No hit | – |
| MB37870 | Hypothetical protein (PHAVU_001G251600g) mRNA | XM_007163580.1 | Myb-like protein A-like (LOC102668250) | XM_006603030.1 |
| MB21522 | Hypothetical protein (PHAVU_001G003400g) mRNA | XM_007160567.1 | E3 ubiquitin-protein ligase ATL23-like (LOC100817919) | XM_003544968.2 |
| MB51985 | Hypothetical protein (PHAVU_001G019300g) mRNA | XM_007160758.1 | No hit | – |
| MB13673 | Hypothetical protein (PHAVU_011G168000g) mRNA | XM_007133238.1 | Uncharacterized LOC100789939 (LOC100789939) | XM_003546588.2 |
| MB34120 | Hypothetical protein (PHAVU_007G247100g) mRNA | XM_007145478.1 | Uncharacterized LOC100806623 (LOC100806623) | XM_006588868.1 |
| MB15445 | Hypothetical protein (PHAVU_005G153500g) mRNA | XM_007150376.1 | Salt tolerance protein-like (LOC100814727) | NM_001255495.2 |
| MB79303 | Hypothetical protein (PHAVU_007G266900g) mRNA | XM_007145716.1 | RING-H2 finger protein ATL70-like (LOC100806773) | XM_003519059.2 |
| MB24478 | Hypothetical protein (PHAVU_007G234900g) mRNA | XM_007145328.1 | Pentatricopeptide repeat-containing protein At5g04810, chloroplastic-like (LOC100784052) | XM_003556830.2 |
| MB8236 | Hypothetical protein (PHAVU_005G132700g) mRNA | XM_007150110.1 | BEL1-like homeodomain protein 2-like (LOC100790583) | XM_003539377.2 |
| MB22067 | Hypothetical protein (PHAVU_006G195000g) mRNA | XM_007148218.1 | Transcription factor bHLH112-like (LOC100805087) | XM_003541714.2 |
| MB11659 | 5'-adenylylsulfate reductase-like protein mRNA | KF033243.1 | 5'-adenylylsulfate reductase-like 5-like (LOC100813728) | NM_001255598.1 |
| MB29754 | Hypothetical protein (PHAVU_008G124100g) mRNA | XM_007140513.1 | CLAVATA3/ESR (CLE)-related protein TDIF-like (LOC100789083) | XM_003532250.2 |
| MB17985 | Hypothetical protein (PHAVU_001G089100g) mRNA | XM_007161614.1 | Transcription factor TCP20-like (LOC100800779) | XM_003553901.2 |
| MB25181 | Hypothetical protein (PHAVU_005G180200g) mRNA | XM_007150721.1 | Mediator of RNA polymerase II transcription subunit 15a-like (LOC100783762) | XM_003547575.2 |
| MB19286 | Hypothetical protein (PHAVU_006G166600g) mRNA | XM_007147867.1 | Transcription factor TCP2-like (LOC100787772) | XM_006594450.1 |
| MB24843 | Hypothetical protein (PHAVU_003G008100g) mRNA | XM_007153055.1 | Zinc finger protein JACKDAW-like (LOC100806198) | XM_006583622.1 |
| MB22568 | Hypothetical protein (PHAVU_002G102600g) mRNA | XM_007157777.1 | Probablemethyltransferase PMT5-like (LOC100812239) | XM_006583887.1 |
| MB25254 | Hypothetical protein (PHAVU_008G254500g) mRNA | XM_007142061.1 | GATA transcription factor 28-like (LOC100812262) | XR_415584.1 |
| MB15212 | No hit | – | No hit | – |
| MB25564 | Hypothetical protein (PHAVU_002G028100g) mRNA | XM_007156856.1 | Zinc finger protein MAGPIE-like (LOC100806404) | XM_003517191.2 |
